# Piezo1-mediated mechanotransduction enhances macrophage oxidized low-density lipoprotein uptake and atherogenesis

**DOI:** 10.1093/pnasnexus/pgae436

**Published:** 2024-10-04

**Authors:** Hamza Atcha, Daanish Kulkarni, Vijaykumar S Meli, Praveen Krishna Veerasubramanian, Yuchun Wang, Michael D Cahalan, Medha M Pathak, Wendy F Liu

**Affiliations:** Department of Bioengineering, University of California, San Diego, La Jolla 92093, USA; Sanford Consortium for Regenerative Medicine, La Jolla 92037, USA; Department of Biomedical Engineering, University of California, Irvine, Irvine 92697, USA; The Edwards Lifesciences Center for Advanced Cardiovascular Technology, University of California, Irvine, Irvine 92697, USA; Department of Biomedical Engineering, University of California, Irvine, Irvine 92697, USA; The Edwards Lifesciences Center for Advanced Cardiovascular Technology, University of California, Irvine, Irvine 92697, USA; Department of Chemical and Biomolecular Engineering, University of California, Irvine, Irvine 92697, USA; Department of Biomedical Engineering, University of California, Irvine, Irvine 92697, USA; The Edwards Lifesciences Center for Advanced Cardiovascular Technology, University of California, Irvine, Irvine 92697, USA; Department of Biomedical Engineering, University of California, Irvine, Irvine 92697, USA; The Edwards Lifesciences Center for Advanced Cardiovascular Technology, University of California, Irvine, Irvine 92697, USA; Department of Physiology and Biophysics, University of California Irvine, Irvine 92697, USA; Department of Biomedical Engineering, University of California, Irvine, Irvine 92697, USA; Department of Physiology and Biophysics, University of California Irvine, Irvine 92697, USA; Sue and Bill Gross Stem Cell Research Center, University of California, Irvine, Irvine 92697, USA; Department of Biomedical Engineering, University of California, Irvine, Irvine 92697, USA; The Edwards Lifesciences Center for Advanced Cardiovascular Technology, University of California, Irvine, Irvine 92697, USA; Department of Chemical and Biomolecular Engineering, University of California, Irvine, Irvine 92697, USA; Department of Molecular Biology and Biochemistry, University of California, Irvine, Irvine 92697, USA

**Keywords:** macrophage, mechanotransduction, Piezo1, atherosclerosis, foam cell

## Abstract

Macrophages in the vascular wall ingest and clear lipids, but abundant lipid accumulation leads to foam cell formation and atherosclerosis, a pathological condition often characterized by tissue stiffening. While the role of biochemical stimuli in the modulation of macrophage function is well studied, the role of biophysical cues and the molecules involved in mechanosensation are less well understood. Here, we use genetic and pharmacological tools to show extracellular oxidized low-density lipoproteins (oxLDLs) stimulate Ca^2+^ signaling through activation of the mechanically gated ion channel Piezo1. Moreover, macrophage Piezo1 expression is critical in the transduction of environmental stiffness and channel deletion suppresses, whereas a gain-of-function mutation exacerbates oxLDL uptake. Additionally, we find that depletion of myeloid Piezo1 protects from atherosclerotic plaque formation in vivo. Together, our study highlights an important role for Piezo1 and its respective mutations in macrophage mechanosensing, lipid uptake, and cardiovascular disease.

## Introduction

Macrophages are mechanosensitive cells of the innate immune system that are central regulators of atherosclerosis and cardiovascular disease. These innate immune cells are recruited to the arterial wall, where they are responsible for the ingestion and removal of circulating lipids such as oxidized low-density lipoproteins (oxLDLs) (1). Uptake of oxLDL in macrophages is largely controlled by scavenger receptors including CD36 and SRA1 (2), which recognize and bind oxLDL, apoptotic cells, glycated proteins, and amyloid-forming peptides (3, 4). Moreover, the expression of these receptors is regulated by exposure to lipids as well as their transport and metabolism within cells. Elevated plasma cholesterol and inefficient systemic clearance results in enhanced cholesterol uptake and the formation of foam cells that are rich in lipid droplets. Continued and excessive cholesterol loading triggers apoptosis of foam cells initiating the development of a necrotic core in an atherosclerotic plaque. Plaque formation also results in deposition of abundant extracellular matrix proteins, including fibronectin, and is associated with stiffening of the arterial microenvironment (5–7). While it is well appreciated that disease alters tissue mechanics, the role of mechanical cues and mechanosensitive molecules in regulating macrophage function and lipid uptake in atherosclerosis remains understudied.

The mechanosensitive ion channel Piezo1 has recently been shown to play a major role in macrophage function (8), and mutations to this channel are implicated in several diseases (9). Piezo1-specific mutations lead to lymphatic dysplasia (10), which is caused by Piezo1 loss of function (11, 12). Gain-of-function (GOF) point mutations, on the other hand, slow channel inactivation and therefore enhance ion movement through the channel (13). In mice, the R2482H Piezo1 GOF mutations (equivalent to R2456H in humans and affecting 30% of individuals in African populations) increased macrophage phagocytic activity, resulting in compromised iron metabolism and heightened red blood cell turnover (9). Additionally, the GOF mutation was found to reduce parasitemia in human red blood cells in vitro and protect mice from cerebral malaria (14). However, while cardiovascular diseases are also known to be more prevalent within the same racial and ethnic group (15), the role of these mutations in atherosclerosis and cardiovascular disease is unknown.

In this study, we examine the role of Piezo1 in the modulation of macrophage oxLDL uptake and atherosclerotic plaque formation. We utilize genetic mouse models with Piezo1 depletion (*Piezo1^ΔLysM^*) or GOF mutations (*Piezo1^LysM−GOF^*) in myeloid cells and found that oxLDL stimulated Ca^2+^ influx in a Piezo1-dependent manner and that Piezo1 activity enhanced oxLDL uptake in vitro and promoted atherosclerotic plaque formation in vivo. Our study identifies Piezo1 as a critical mechanosensitive molecule involved in foam cell formation as well as atherosclerotic plaque development and progression.

## Results

### Piezo1 depletion reduces Ca^2+^ influx and oxLDL uptake

**Fig. 1.**
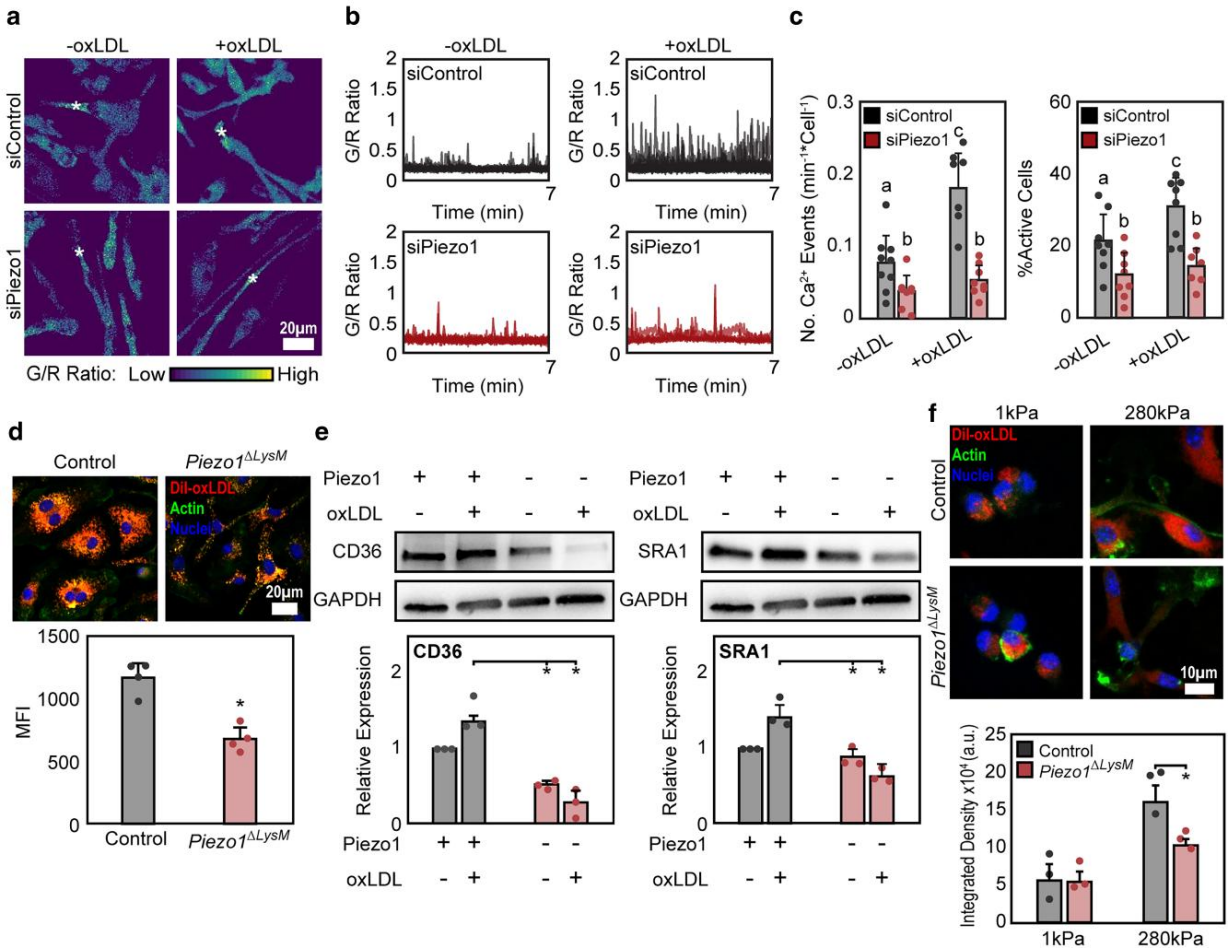
Piezo1 depletion suppresses Ca^2+^ activity and oxLDL uptake. Representative GCaMP6f/tdTomato (G/R) ratio images (a) as well as traces of individual Ca^2+^ events (b), and quantification of number of Ca^2+^ events and fraction of cells showing Ca^2+^ elevations (c), taken from a 7-min time-lapse video of siControl and siPiezo1-treated Salsa6f-expressing bone marrow-derived macrophages (BMDMs) both with and without oxLDL exposure. Asterisks denote the occurrence of a Ca^2+^ event. Data obtained from *n* = 7–8 videos; the letters on the top of graphs indicate statistical significance of *P* < 0.05 among groups as determined by Student's t test. d) Representative images (top) and mean fluorescence intensity quantification (bottom) of oxLDL uptake. e) Representative western blots (top) and quantification (bottom) of CD36 and SRA1 in BMDMs isolated from control and *Piezo1^ΔLysM^* mice following oxLDL treatment. f) Representative images (top) and total fluorescence intensity quantification of oxLDL uptake (bottom) in control and *Piezo1^ΔLysM^* BMDMs cultured on 1 and 280 kPa polyacrylamide hydrogels. The error bars denote mean ± SD for a minimum of 3 independent experiments. *P < 0.05 as determined by Student's t test.

Using siRNA or transgenic mice with channel depletion (16, 17), we first examined the role of Piezo1 in modulating Ca^2+^ events, uptake, stiffness mechanotransduction, and responses to oxLDL. We observed that oxLDL treatment enhanced Ca^2+^ events and that siRNA-mediated Piezo1 knockdown abrogated this increased activity (Fig. 1a–c). Functionally, we found that oxLDL accumulation was reduced in cells lacking Piezo1 (*Piezo1^ΔLysM^*) and consistent with this observation, cells had reduced CD36 and SRA1 uptake receptor expression when treated with oxLDL (Fig. 1d–e). In contrast, control Piezo1-expressing cells increased uptake and expression of receptors in response to oxLDL treatment (Fig. 1e). However, no differences in oxLDL binding to the cell surface were observed between control and Piezo1 lacking macrophages, suggesting that Piezo1 primarily modulates oxLDL internalization. Moreover, given that Piezo1 is a mechanically gated ion channel, which has been shown to sense and transduce a variety of different physical cues, and that atherosclerosis is often associated with stiffening of the arterial microenvironment, we next evaluated the role of substrate stiffness in regulating Piezo1-mediated oxLDL uptake. Atherosclerotic plaque development has been shown to result in localized areas of enhanced stiffness within the artery, with regions measuring ∼250 kPa using atomic force microscopy (18). Therefore, we cultured macrophages on fibronectin-conjugated polyacrylamide hydrogels representing a soft (1 kPa) or stiff (280 kPa) microenvironment and stimulated cells with oxLDL (19). We found that stiffness enhanced oxLDL uptake in control macrophages and that *Piezo1^ΔLysM^* reduced uptake on stiff substrates when compared with control (Fig. 1f). Additionally, significant differences in oxLDL uptake were also observed between Piezo1-deficient cells cultured on 1 and 280 kPa surfaces. Given that ion channel depletion did not fully abrogate increased oxLDL uptake on stiff surfaces, it is plausible that Piezo1-independent mechanisms are also involved (Fig. 1f). Together, our data suggest that Piezo1 is activated in response to oxLDL treatment, and its depletion reduces uptake on stiff surfaces.

### Piezo1 GOF promotes uptake through modulation of oxLDL receptor expression

**Fig. 2.**
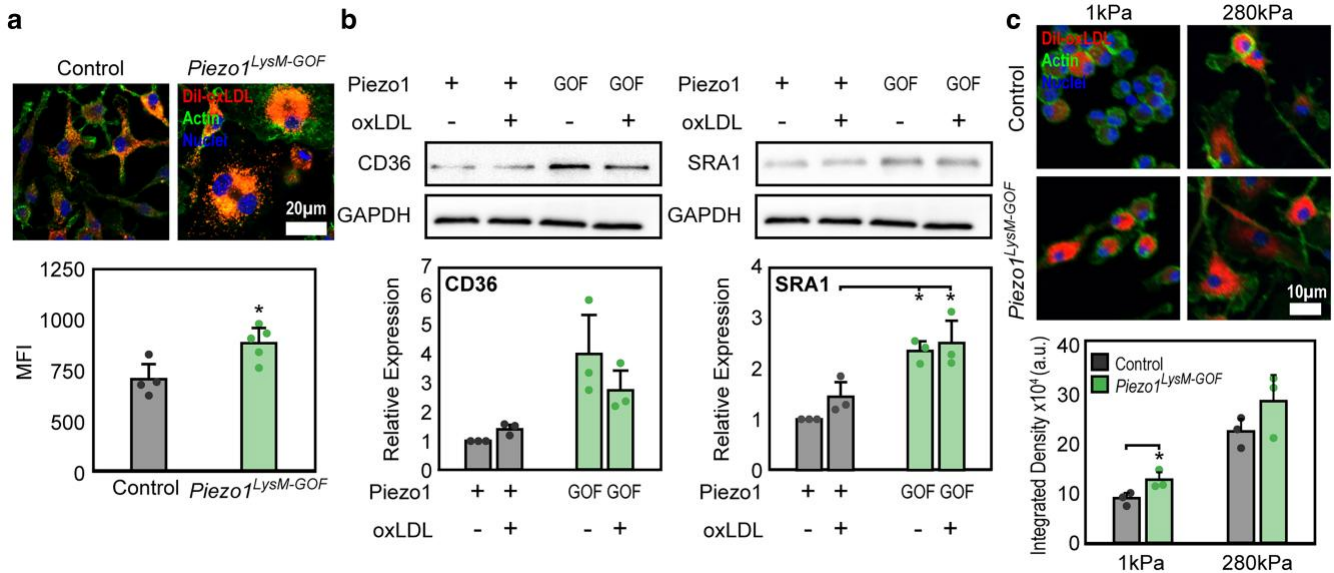
Piezo1 GOF enhances oxLDL uptake. a) Representative images (top) and mean fluorescence intensity quantification of oxLDL uptake (bottom) in BMDMs isolated from control and *Piezo1^GOF^* mice. b) Representative western blots (top) and quantification (bottom) of CD36 and SRA1 in BMDMs isolated from control and *Piezo1^GOF^* mice following oxLDL treatment. c) Representative images (top) and mean fluorescence intensity quantification of oxLDL uptake over time (bottom) in BMDMs isolated from control and *Piezo1^GOF^* mice. The error bars denote mean ± SD for a minimum of 3 independent experiments. *P < 0.05 as determined by Student's t test.

In contrast to Piezo1 depletion, Piezo1 GOF (*Piezo1^LysM−GOF^*) exhibited enhanced oxLDL uptake compared with control cells and showed increased SRA1 uptake receptor expression, both with and without oxLDL stimulation (Fig. 2a and b). *Piezo1^LysM−GOF^* also exhibited enhanced uptake on soft surfaces when compared with control cells (Fig. 2c). Together, our data suggest an important role for Piezo1 in the modulation of oxLDL uptake, with Piezo1 GOF positively associated with uptake receptor expression and resulting in oxLDL accumulation within cells. When combined, our data suggest that Piezo1 GOF mutation enhances oxLDL uptake through increases in uptake receptor expression and can promote mechanotransduction on soft surfaces.

### Piezo1 is highly expressed within and enhances atherosclerotic plaque development

**Fig. 3.**
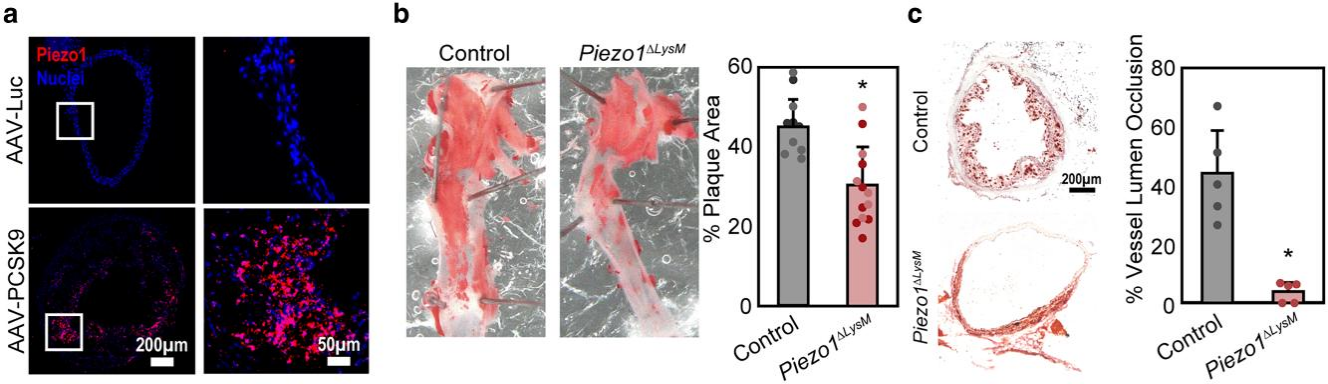
Piezo1 enhances atherosclerotic plaque formation in vivo. a) Representative RNAscope images of *Piezo1* in a cross-section of the aortic arch in control AAV-Luc and AAV-PCSK9-treated mice following a 3-month high-fat diet. b) Representative en-face images of aortas isolated from control and *Piezo1^ΔLysM^* mice stained with Oil Red O (ORO) (left) and analysis of percent plaque area across surface of the aorta (right). The darker data points denote male mice, while the lighter data points denote female mice. The error bars denote mean ± SD for 12 independent experiments. *P < 0.05 as determined by Student's t test. c) A representative image of histology sections from isolated aortas stained with ORO (left) and analysis of percent vessel lumen occlusion. The error bars denote mean ± SD for 5 independent experiments. *P < 0.05 as determined by Student's t test.

Finally, we evaluated the role of Piezo1 in modulating atherosclerosis in vivo using a mouse model (20). We used liver-targeted adenoviral overexpression of murine proprotein convertase subtilisin/ kexin type 9 (PCSK9), which increases the degradation of LDLR and also regulates triglycerides in the small intestine and modulates megalin-driven protein reabsorption in the kidney (21–23). When combined with a high-fat diet, PCSK9 elevates systemic cholesterol levels in mice, resulting in atherosclerotic plaque formation (22, 24). Following 3 months on a high-fat diet, we observed enhanced *Piezo1* gene expression localized to atherosclerotic plaques within adeno-associated virus (AAV)-PCSK9-treated mice when compared with control, suggesting a potential role for Piezo1 in plaque development and progression in vivo (Fig. 3a). We treated control and *Piezo1^ΔLysM^* mice with AAV-PCSK9 and found that *Piezo1^ΔLysM^* mice had reduced plaque formation when compared with control mice expressing Piezo1 (Fig. 3b and c). Interestingly, while both en-face and histological staining indicate a significant reduction in plaque formation, histology sections suggest a more profound effect on plaque development, as measured by reduced vessel closure in mice lacking myeloid Piezo1 (Fig. 3c). Together, the data presented in this study provide key insights into the role of Piezo1 in the mechanosensation of stiffness as well as the modulation of oxLDL uptake and atherosclerotic plaque formation.

## Discussion

Our study suggests an important role for Piezo1 in oxLDL uptake and atherosclerosis. While other channels, such as TRPM7 and Orai1, have been shown to regulate Ca^2+^ activity in response to oxLDL as well as inflammatory stimuli in macrophages (25, 26), our data show that Piezo1 is required for Ca^2+^ influx in response to oxLDL stimulation. The molecular mediators responsible for atherosclerosis are still being elucidated; however, shear stress and stiff environments have been shown to enhance oxLDL uptake (27, 28). Recent studies have uncovered the importance of Piezo1 in regulating cell morphology and mechanotransduction pathways across a variety of developmental and pathological conditions involving macrophages (8, 16). We show that Piezo1 plays a pivotal role in sensing stiff environments, modulating the expression of key uptake transporters, enhancing oxLDL accumulation, and leading to macrophage foam cell formation in vitro and the development and progression of atherosclerosis in vivo. Interestingly, while it is known that oxLDL transporter expression is tightly regulated through positive feedback mechanisms involving a number of different signaling pathways such as NFkB or PPARγ, our data suggest that Piezo1 may be critical to and enhances these processes (29–31).

Our work also utilizes genetic tools to evaluate the role of Piezo1 mutations in oxLDL uptake and atherosclerosis. Our data suggests that *Piezo1^LysM−GOF^* enhances macrophage oxLDL uptake and receptor expression. These Piezo1 GOF mutations are known to be prevalent in ∼30% of individuals of African descent (14). This also correlates with increased prevalence and risk of cardiovascular disease among individuals of the same ethnicity (15, 32), although no direct link has thus far been established. It is plausible that while socioeconomic, underlying disease, and lifestyle are often implicated, genetic mutations to the Piezo1 channel could also contribute as a risk factor in cardiovascular disease. In contrast, *Piezo1^ΔLysM^* reduces uptake and suppresses atherosclerotic plaque formation within mice. Our study, combined with recent findings highlighting the role of GOF mutations in murine cardiac hypertrophy and fibrosis (33), suggests that the development of Piezo1-specific inhibitors could potentially reduce atherosclerotic plaque development and may also help alleviate other cardiovascular diseases. However, further studies will be needed to show whether Piezo1 and its respective mutations provide additional risk for disease in humans. Moreover, stiffness measurements, characterization of receptor expression of explanted aortas and in vivo cholesterol modulation, as well as detailed mechanistic studies will provide more insight into Piezo1-mediated molecular pathways involved in cardiovascular disease.

## Materials and methods

All animal experiments were approved by the University of California, Irvine's Institutional Animal Care and Use Committee under protocol no. AUP-20-047. Extended methods are provided in the Supplemental Information.

## Supplementary Material

pgae436_Supplementary_Data

## Data Availability

All data supporting the key findings of this study are available within the article and its Supplementary Information files. Extended methods are given in Supplementary Information.
