# Supplementary material for: Piezo1-mediated mechanotransduction enhances macrophage oxidized low-density lipoprotein uptake and atherogenesis
Source: PNAS Nexus. 2024 Oct 4;3(11):pgae436. doi: 10.1093/pnasnexus/pgae436 (PMC11563038; doi:10.1093/pnasnexus/pgae436)
Supplement: pgae436_Supplementary_Data [file pgae436_supplementary_data.docx]

Supplemental Information for:

**Piezo1-mediated mechanotransduction enhances macrophage oxLDL uptake and atherogenesis**

Hamza Atcha^1,2^, Daanish Kulkarni^3,4^, Vijaykumar S. Meli^3,4,5^, Praveen Krishna Veerasubramanian^3,4^, Yuchun Wang^3,4^, Michael D. Cahalan^6^, Medha M. Pathak^3,6,7^, and Wendy F. Liu^3,4,5,8^

^1^ Department of Bioengineering, University of California, San Diego, La Jolla, USA

^2^ Sanford Consortium for Regenerative Medicine, La Jolla, USA

^3^ Department of Biomedical Engineering, University of California, Irvine, Irvine, USA

^4^ The Edwards Lifesciences Center for Advanced Cardiovascular Technology, University of California, Irvine, Irvine, USA

^5^ Department of Chemical and Biomolecular Engineering, University of California, Irvine, Irvine, USA

^6^ Department of Physiology and Biophysics, University of California Irvine, Irvine, USA

^7^ Sue and Bill Gross Stem Cell Research Center, University of California, Irvine, Irvine, USA

^8^ Department of Molecular Biology and Biochemistry, University of California, Irvine, Irvine, USA

Correspondence should be addressed to W.F.L. (email:[wendy.liu@uci.edu](about:blank))

**Extended Methods**

| **Antibody** | **Vendor, Cat. #** | **Application** | **Dilution** |
| --- | --- | --- | --- |
| CD36 | ProteinTech, 18836-1-AP | WB | 1:2000 |
| SRA1 | ProteinTech, 24655-1-AP | WB | 1:2000 |
| GAPDH | Santa Cruz Biotechnology, sc-59540 | WB | 1:2000 |

**Animals.** Generation of *Piezo1^ΔLysM^* mice was accomplished through breeding Piezo1^flox/flox^ (Jackson Laboratories stock no. 029213) and LysM^Cre/Cre^ (Jackson Laboratories stock no. 004781) mice together to generate progeny that were heterozygous for both genes. The generated heterozygotes were then bred with Piezo1^flox/flox^ mice to generate Piezo1^flox/flox^LysM^Cre/+^ (*Piezo1^ΔLysM^*) and Piezo1^flox/+^LysM^Cre/+^ (*Piezo1^fl/+^*) mice. A similar breeding scheme was utilized to generate LSL-Salsa6f-LysM1^Cre/+^ mice used for calcium imaging. Wild type C57BL/6J mice (Jackson Laboratories) were also used in experiments that did not require genetic manipulation. Piezo1^GOF/+^LysM^Cre/+^ (Piezo1^LysM-GOF^) mice and Piezo1^GOF/+^controls were provided by the Patapoutian lab. Piezo1^LysM-GOF^ mice contain a Piezo1 point mutation (R2482H; equivalent to the R2456H mutation commonly found in the African population) that is known to slow inactivation allowing for the channel to be open for longer (1). Experiments were performed using animals of similar age and littermates were used as controls. Mice were maintained at 12 hr light/dark cycles within temperature (70-74°F) and humidity (30-70%) controlled rooms.

**Cell isolation and culture.** Bone marrow derived macrophages (BMDMs) were harvested from the femurs of 6-12-week-old C57BL/6J mice. Bone marrow cells were collected by flushing the bone marrow of the femur with DMEM supplemented with 10% heat-inactivated FBS, 2mM l-glutamine, 1% penicillin/streptomycin (all from Thermo Fisher), and a 10% conditioned media produced from CMG 14-12 cells expressing recombinant mouse macrophage colony stimulating factor (MCSF), which induces differentiation of bone marrow cells to macrophages. To remove red blood cells, the collected bone marrow cells were treated with a red cell lysis buffer, and then centrifuged before being resuspended in the previously mentioned media. After 7 days, the cells were harvested using an enzyme-free dissociation buffer (Fisher Scientific) and seeded onto surfaces that were coated with a 10µg/mL fibronectin (Corning) solution. BMDMs were seeded at a density of ~3.9 x 10^4^ cells/cm^2^ and were incubated overnight prior to stimulation with media or 5µg/mL Human high-oxidized LDL (Kalen Biomedical Cat # 770252-7) or DiI conjugated human high-oxidized LDL (Kalen Biomedical Cat # 770262-9). Following stimulation, cells were incubated for 24 h prior to collection.

**oxLDL binding assay**. Established protocols were used to evaluate the effects of Piezo1 on oxLDL binding to the surface of macrophages (2). Briefly, control and Piezo1 depleted macrophages were cultured with 5µg/mL of oxLDL on ice for a period of 2 hrs prior to fixation and imaging. The mean fluorescence intensity was calculated as 202.00±23.30 and 198.42±31.48 for control and *Piezo1^ΔLysM^* macrophages, respectively (N = 100 cells).

**Polyacrylamide gel fabrication.** Polyacrylamide hydrogels of varying stiffness were fabricated using protocols previously described (3). Briefly, cover slips were cleaned with 70% ethanol and dried prior to 10 min UVO treatment. The cover slips were then treated with bind-silane (solution containing 95% of 95% ethanol, 0.3% 3-(Trimethoxysilyl) propylmethacrylate, and 5% of 10% acetic acid) and were incubated for 5 mins at room temperature. The coverslips were then washed with ethanol prior to incubation at 70°C for one hour. Meanwhile, glass slides were treated with silanization solution I and incubated in a vacuum desiccator for 5 mins. Glass slides were washed with DI water and blotted dry prior to gel formation. Solutions containing varying ratios of acrylamide:bis-acrylamide were pipetted onto the glass slides and the coverslip was placed onto the solution such that the polyacrylamide gel would be sandwiched between the bind-silane treated coverslip and salinization solution I treated glass slide. Gels were allowed to polymerize for 30 mins and were then removed from the glass slide and placed into culture plates. The resulting hydrogels were conjugated with 20 µg/ml of fibronectin using sulfo-SANPAH (Thermo Scientific) overnight at 4°C.

**Polyacrylamide gel characterization.** Parallel plate rheometry (Anton Paar) was used to characterize the stiffness of the fabricated gels. Briefly, gel solutions were mixed and allowed to gel on the rheometer stage. Time sweeps were performed from which storage and loss moduli were calculated. The elastic modulus was also calculated and found to be 0.91±0.02 kPa and 257.50±9.50 kPa for the soft and stiff conditions, respectively, across two independent replicates.

**Ca^2+^ imaging and analysis.**

Confocal Ca^2+^ imaging studies were performed as previously described (4). Briefly, BMDMs from LSL-Salsa6f-LysM^Cre/+^ mice were seeded on fibronectin coated 35mm MatTek dishes. Confocal imaging of Ca^2+^ dynamics in Salsa6f macrophages was accomplished using an Olympus Fluoview FV3000RS confocal laser scanning microscope which is equipped with a high-speed resonance scanner and IX3-ZDC2 Z-drift compensator. Cells were maintained at 37°C using the Tokai Hit incubation stage, excited using sequential line scan at 488nm and 561nm and imaged using an Olympus 40x silicone oil objective (NA 1.25). Ratiometric analysis of Ca^2+^ signals were performed through using ImageJ software. The number of events were computed through the use of a MATLAB script, previously described (4). Briefly, a polynomial fit was used to compute and subtract baseline values from G/R ratios over time. This was followed by using a Gaussian filter to smooth the data before using a MATLAB peak finding function to identify the number of peaks within a signal, which corresponds to the number of Ca^2+^ events. Additionally, the total % active cells was obtained by dividing cells with one or more Ca^2+^ events to the total number of cells present in the field of view.

**RNA interference.** For experiments involving the reduction of *Piezo1* expression, unstimulated macrophages were transfected with non-target or PIEZO1 siRNA (both Dharmacon) in a Nucleofector® solution obtained from a primary cell 4D-Nucleofector® kit (Lonza), as previously described (5). Following transfection, the cells were supplemented with warm media before being seeded onto experimental substrates. The transfected cells were allowed to adhere for 72 hours prior to oxLDL stimulation.

**Western blotting.** BMDMs were rinsed with PBS before being exposed to a lysis buffer, a combination of RIPA lysis buffer and 1% protease inhibitor (both from Fisher Scientific). The substrates were scraped to release the adhered cells and the lysate was collected. The lysate was spun at 16000g for 15 minutes and the supernatant was obtained. The proteins were denatured through the use of a Laemmli buffer supplemented with 5% 2-mercaptoethanol at 95°C for 10 minutes before each sample was loaded into a well of a 4-15% mini-PROTEAN^TM^ precast gel (all from Biorad). Gel electrophoresis resulted in the separation of proteins before being transferred onto nitrocellulose membranes using the iBlot dry blotting system (Thermo Fisher Scientific). Following electroblotting, the membranes were blocked using 5% nonfat milk in TBST overnight at 4°C. After 30 minutes of washing in TBST, the membranes were probed with either a CD36 (Proteintech - 18836-1-AP), SRA1 (Proteintech - 24655-1-AP), or GAPDH (Santa Cruz Biotechnology - sc-59540) primary antibody at a 1:2000 dilution for 1 hour at room temperature. An additional 30 minutes of washing in TBST followed before the membranes were probed with secondary antibodies at 1:2000 dilution at room temperature for 1 hour. The membrane was then washed in TBST and immersed into a chemiluminescent HRP substrate solution (Thermo Scientific) and imaged using a ChemiDoc XRS System (Biorad) which normalizes the signal in each blot to the highest expressing condition.

**Immunofluorescence.** Following stimulation, BMDMs were fixed in 4% paraformaldehyde for a period of 10 mins. The fixed cells were washed in PBS prior to permeabilization in 0.1% Triton-X in PBS. Following additional PBS washes the cells were incubated with Alexa Fluor 488 phalloidin (Fisher Scientific), diluted 1:100 in PBS, and Hoechst (Invitrogen), diluted 1:2000 in PBS, for 30 minutes at room temperature. The cells were thoroughly washed with PBS, before being mounted onto a glass slide and imaged using a Zeiss LSM700 confocal microscope or an Olympus Fluoview FV3000 confocal laser scanning microscope. Laser settings were adjusted to the condition with highest intensity. Approximately 50 cells in each condition were outlined per experiment and the mean intensity or total intensity was computed for each cell using ImageJ.

**Murine atherosclerosis model.** Liver promoter driven adenoviral-mediated overexpression of PCSK9 (AAV-PCSK9) and murine atherosclerotic plaque development was accomplished as previously described (6, 7). PCSK9 is a small protein expressed and secreted within the liver and is responsible for the degradation of LDLR (6, 7). Therefore, PCSK9 overexpression reduces LDLR abundance and enhances systemic cholesterol levels, a condition required for atherosclerotic plaque formation in mice (8). Plasmids used for AAV model include Addgene item IDs: 112867 (pAd/DeltaF6), 58376 (pAAV/D377Y-mPCSK9), 112865 (pAAV2/9n). Briefly, AAV-PCSK9 was administered through tail vein injections in a minimum of 6 control and 6 *Piezo1^ΔLysM^* for both male and female mice at 1 x 10^11^ VG. The mice were subsequently fed a high fat diet (16% fat and 1.25% cholesterol, Research Diets, cat # D12336) for a period of three months prior to isolation of aortas. Mice treated with AAV-PCSK9 were observed to have 33.47±6.95% plaque area in their aortic arch compared to 3.09±1.85% for control untreated mice (N = 5).

***In situ* hybridization (ISH).** ISH for *Piezo1* expression was performed using the RNAscope Multiplex Fluorescent Reagent Kit vs (#322000; ACD Bio) using the manufacturer’s protocol for fresh-frozen tissue sections. Briefly, aortas from AAV-PCSK9 and control AAV-Luc mice that were fed a high fat diet for three months were isolated, embedded in OCT, and sectioned. Sections were taken from the aortic arch and fixed with 4% paraformaldehyde for 15 min which was followed by a PBS wash and dehydration in 50%, 70%, and 100% ethanol for 5 min each. In preparation for RNAscope ISH, the sections were treated with RNAscope Hydrogen Peroxide for 10 min followed by RNAscope Protease IV for 30 min. For the ISH assay, tissue sections were incubated with the *Piezo1* probe (#400181; ACD Bio). Following hybridization, the probe was subsequently tagged with the Opal 570 reagent pack (FP1488001KT; Akoya Bio). Cell nuclei were counterstained with DAPI for 30 sec and mounted with Fluoromount-G (100241-874; VWR). Fluorescence imaging was performed on the Keyence BZ-X810 Widefield Microscope at the UCI Stem Cell Research Center.

**En face and histology analysis.** For en-face analysis, whole aortas were isolated from mice following three months on a high fat diet, cleared of surrounding fat and connective tissue under a dissecting microscope, and fixed using a 4% paraformaldehyde solution for 15 min which was followed by PBS washes. The resulting tissue was longitudinally split to expose the endothelium, rinsed in 60% isopropanol, and stained with an oil red o solution for 20 min. Tissue was subsequently washed with isopropanol and water prior to being pinned onto a paraffin wax block and imaged. For histology, the tissue surrounding the aortic arch was isolated, cleaned, embedded in OCT and frozen at -80°C. The aortic arch was sectioned, fixed, and stained and imaged as described above. ImageJ was used to calculate % vessel lumen obstruction.

**Statistics and reproducibility.** Data are presented as the mean ± standard deviation across at least three independent experiments. Representative images are accompanied with quantification from a minimum of three independent experiments. Comparisons were performed using a two-tailed Student’s t-test, two-tailed paired t-test, or two-tailed Mann-Whitney U test, as indicated in figure legends, and **p* < 0.05 was considered significant.

**References**

1. S. Ma, *et al.*, Common PIEZO1 Allele in African Populations Causes RBC Dehydration and Attenuates Plasmodium Infection. *Cell* **173**, 443-455.e12 (2018).

2. R. Goswami, *et al.*, TRPV4 calcium-permeable channel is a novel regulator of oxidized LDL-induced macrophage foam cell formation. *Free Radic. Biol. Med.* **110**, 142–150 (2017).

3. J. R. Tse, A. J. Engler, Preparation of Hydrogel Substrates with Tunable Mechanical Properties. *Current Protocols in Cell Biology* **47**, 10.16.1-10.16.16 (2010).

4. H. Atcha, *et al.*, Mechanically activated ion channel Piezo1 modulates macrophage polarization and stiffness sensing. *Nat Commun* **12**, 3256 (2021).

5. H. Atcha, *et al.*, Crosstalk Between CD11b and Piezo1 Mediates Macrophage Responses to Mechanical Cues. *Frontiers in Immunology* **12**, 3505 (2021).

6. C. Goettsch, *et al.*, A single injection of gain-of-function mutant PCSK9 adeno-associated virus vector induces cardiovascular calcification in mice with no genetic modification. *Atherosclerosis* **251**, 109–118 (2016).

7. S. Kumar, D.-W. Kang, A. Rezvan, H. Jo, Accelerated atherosclerosis development in C57Bl6 mice by overexpressing AAV-mediated PCSK9 and partial carotid ligation. *Laboratory Investigation* **97**, 935–945 (2017).

8. Y. T. Lee, *et al.*, Mouse models of atherosclerosis: a historical perspective and recent advances. *Lipids Health Dis* **16** (2017).
